# Advances in Isorhamnetin Treatment of Malignant Tumors: Mechanisms and Applications

**DOI:** 10.3390/nu17111853

**Published:** 2025-05-29

**Authors:** Chen Mei, Ying Liu, Xueze Lyu, Zhaoling Jiang, Zhenyi Liu, Yan Zhi, Xiaolong Xu, Hongjun Wang

**Affiliations:** 1 Institute of Animal Husbandry and Veterinary Medicine, Beijing Academy of Agriculture and Forestry Sciences, Beijing 100097, China; meichen@baafs.net.cn (C.M.);; 2 College of Veterinary Medicine, China Agricultural University, Beijing 100193, China; 3 Beijing General Animal Husbandry Station, Beijing 100107, China; 4 Laboratory for Clinical Medicine, Capital Medical University, Beijing 100010, China

**Keywords:** isorhamnetin, malignant tumor, antitumor mechanism, clinical application, natural medicine

## Abstract

Isorhamnetin (ISO) is a natural flavonoid compound that has become a main research topic in recent years due to its multitargeted antitumor properties. In this paper, we systematically review the molecular basis of the inhibition of malignant tumors by ISO, including through the regulation of the cell cycle, PI3K/AKT/mTOR pathway, MAPK pathway, apoptosis/autophagy-related pathways, and the tumor microenvironment. We also explore its synergistic effects with chemotherapy/targeted therapies and its potential for clinical translation. Experimental studies have shown that ISO can not only directly inhibit tumor proliferation by inducing tumor cell cycle arrest, mitochondria-dependent apoptosis, and endoplasmic reticulum stress, but also enhance antitumor immune responses by regulating the immune microenvironment. Pharmacokinetic studies have shown that novel delivery systems, such as nano-formulations, significantly enhance the bioavailability of ISO. Notably, ISO has demonstrated unique advantages in attenuating the nephrotoxicity of chemotherapeutic agents, protecting normal cells, and reversing tumor resistance. However, the optimal dosing regimen, dose–effect relationship, and cross-species applicability need to be further validated by large-scale preclinical animal experiments and clinical trials. This paper provides a theoretical basis for the development and application of ISO for the treatment of malignant tumors and highlights its potential value in animal models.

## 1. Introduction

As of 2024, the global cancer burden continues to increase and has become one of the primary non-communicable diseases threatening public health. Occupational exposure to carcinogens, infections from pathogens such as Helicobacter pylori and human papillomavirus (HPV), as well as metabolic abnormalities, all play significant roles in elevating the risk of disease. At the same time, the landscape of cancer is experiencing a dual transformation: while traditional high-incidence tumors of the digestive system continue to pose significant challenges, there is also a notable increase in lifestyle-related adenocarcinomas and tumors among younger populations. For example, breast cancer shows a significantly higher incidence in the female population, while the incidence of lung cancer is significantly and positively correlated with a history of smoking and exposure to environmental pollution. For example, breast cancer is showing a significantly higher incidence in the female population, while the incidence of lung cancer is significantly and positively correlated with a history of smoking and exposure to environmental pollution. Meanwhile, the incidence of malignant tumors of the digestive system, especially colorectal and gastric cancers, continues to rise, and their development is closely related to an unbalanced dietary structure, high-fat and low-fiber dietary patterns, and other poor dietary habits [1]. It is noteworthy that the incidence of liver cancer caused by hepatitis B virus, hepatitis C virus, and alcohol abuse remains high in specific geographic regions [2]. Those malignant tumors are generally characterized by atypical early clinical symptoms and a high degree of invisibility, resulting in the progression to intermediate and advanced stages at the time of clinical diagnosis, which is an important reason for the persistently high rate of cancer-related mortality. In addition, cancer not only presents a serious threat to patients’ lives and health but also imposes a heavy economic burden on patients’ families and the social healthcare system due to high treatment costs and long-term care requirements [3,4].

The three pillars of malignant tumor treatment, surgery, chemotherapy, and radiotherapy dominate in clinical practice [5]. However, there are significant limitations to the traditional treatment modes. Although radiotherapy has the advantage of molecular targeting, its clinical application rate exhibits significant geographical differences. Chemotherapeutic drugs often lead to serious adverse effects such as bone marrow suppression and organ function damage due to broad-spectrum cytotoxic effects. About 30% of tumor patients are forced to terminate treatment because they cannot tolerate the toxic side effects of traditional treatments [6], thus highlighting the urgent need for the development of new low-toxicity treatments.

In recent years, with breakthroughs in tumor immunotherapy, immune checkpoint inhibitors have made revolutionary progress. Recently, lysoviral therapy has also emerged as a novel biotherapeutic strategy that specifically lyses tumor cells while activating antitumor immune responses through genetic engineering or the screening of natural viral strains. Approximately 60% of anticancer drugs are derived from natural products or their derivatives, and such bioactive compounds exert anticancer effects through mechanisms including cell cycle modulation and apoptosis [7]. Plant-derived terpenoids, alkaloids, and polyphenols have gradually advanced from experimental studies to the clinical translation stage, providing an important breakthrough for the development of highly effective and low-toxicity alternatives to chemotherapies.

Flavonoids are a class of natural products with significant biological activities and that contain flavonoid parent nuclei in their chemical structures. Studies have shown that such compounds exhibit multiple advantages in the field of cancer therapy, including their multitarget mechanisms of action that simultaneously regulate multiple signaling pathways, effectively inhibit the proliferation, invasion, and metastasis of tumor cells, and regulate the tumor microenvironment. Compared to traditional chemotherapeutic drugs, flavonoids have lower cytotoxicity and better biocompatibility, which can significantly reduce the damage to normal tissues, thus improving patients’ treatment tolerance and quality of life.

From a drug development perspective, the multitargeting properties of flavonoids can effectively reduce the risk of drug resistance in tumor cells, a feature that gives them a unique advantage in long-term anticancer therapy. In addition, such compounds are present in a variety of biological sources such as plants, microorganisms, and marine organisms, providing a rich material basis for new drug development. Notably, flavonoids can produce synergistic effects with conventional chemotherapies, radiotherapies, and other therapies to improve their therapeutic effects and overcome the limitations of single therapies via combination strategies. Thus, flavonoids have become an important source of lead compounds for anticancer drug discovery and development initiatives due to their unique bioactivity profiles and favorable safety profiles, providing new strategies for clinical cancer therapy [8].

Isorhamnetin (ISO) is a flavonoid with important bioactivities, and its main natural sources include the tissues and organs of leaves, flowers, and fruits from sea buckthorn (*Hippophae rhamnoides* L.) and ginkgo biloba (*Ginkgo biloba* L.). In terms of traditional medicinal value, sea buckthorn fruits are effective in strengthening the spleen and eliminating food, relieving cough and phlegm, activating blood circulation, and eliminating blood stasis. Ginkgo biloba is widely used in therapeutic areas such as activating blood circulation and eliminating blood stasis, clearing the blood channels to alleviate pain, astringing the lungs to calm wheezing, and lowering lipid levels. Recent studies have shown that ISO is a key active ingredient in medicinal plants that exert pharmacological effects.

ISO is systematically named 3,5,7,4-tetrahydroxy-3-methoxyflavone, with a molecular formula of C_16_H_12_O_7_ and a molecular weight of 316.26 g/mol. The basic skeleton of this compound is the characteristic flavonoid structure of benzo-gamma-pyrone, which contains four hydroxyl and one methoxy substituent located at the 3 position. The presence of the methoxy group at the 3 position not only endows ISO with unique chemical properties but also significantly enhances its biological activity, especially its antitumor effects. This structure–activity relationship provides an important chemical basis to study the pharmacological mechanism of action of ISO [9].

ISO, a representative flavonoid molecule, is a versatile compound that demonstrates superior biocompatibility compared to conventional chemotherapy drugs and effectively delays the development of drug resistance. It accomplishes this by modulating the cancer cell cycle, triggering apoptosis, and altering the tumor immune microenvironment. The following is elaborated from different aspects.

## 2. Different Effects of ISO

In recent years, it has been found that ISO has a wide range of cardioprotective effects, which are closely related to its antioxidant and anti-apoptotic properties. Specifically, ISO can resist atherosclerosis, protect endothelial cells, and prevent myocardial ischemia, hypertension, hyperglycemia, and thrombosis. ISO also exhibits significant neuroprotective effects, improving neurological function and enhancing cognitive memory, thereby contributing to the prevention and treatment of neurodegenerative diseases [9].

ISO exhibits significant anti-inflammatory effects and can effectively inhibit inflammatory responses. For example, ISO can alleviate acute lung injury and has antituberculosis effects. Studies have shown that ISO can alleviate the epithelial–mesenchymal transition in vivo, a mechanism that involves the regulation of inflammatory mediators, cytokines, and the production of reactive oxygen species (ROS) [10,11].

**Figure 1:**
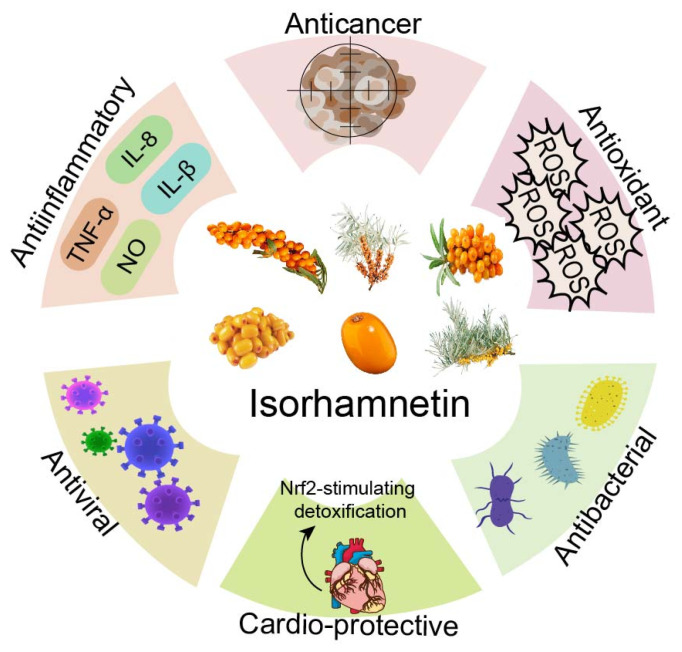
Different effects of ISO.

ISO also possesses antibacterial and antiviral properties. In an infection model of the COVID-19 virus in HEK293 cells, Zhan et al. revealed that ISO inhibited the entry of SARS-CoV-2 spiny pseudotyped virus into angiotensin-converting enzyme 2 (ACE2)-overexpressing cells by targeting the ACE2 protein, thus preventing viral replication [12]. In a coculture system of Staphylococcus aureus and lung cells, topical application of ISO prevented *S. aureus*-induced cellular damage, and thus, ISO may be a key component in the development of antiviral drugs against *S. aureus* infections [13]. In a study of the mechanism of the antibacterial activity of polyphenols, Bhattacharya et al. found that ISO can penetrate bacterial cell membranes through oxidative stress, demonstrating its potential antibacterial activity [14]. The pharmacological effects of ISO and its mechanism of action have become a major area of research. The wide range of bioactivities exhibited by ISO, such as cardioprotective, anti-inflammatory, antibacterial, and antiviral (Figure 1), provides an important theoretical basis for the development of novel therapeutic drugs [15].

## 3. Mechanism of Inhibition of Malignant Tumor Cell Proliferation by ISO In Vitro

### 3.1. ISO Regulates the Cell Cycle to Inhibit Cancer Cell Proliferation

The cell cycle refers to the entire process that cells undergo from the end of one division to the end of the next division. The cell cycle is a key process for cell growth, proliferation, and the maintenance of normal physiological functions. The cell cycle can be divided into two main phases: interphase and the mitotic phase. Interphase is the longest phase of the cell cycle and usually accounts for 90–95% of the entire cell cycle. Interphase is further subdivided into the G1 phase, S phase, and G2 phase. The progression of the cell cycle is tightly regulated by a variety of cell cycle proteins (cyclins) and cell cycle-dependent kinases, which interact with each other to form a complex regulatory network that ensures the orderly progression of the cell cycle. The precise regulation of the cell cycle is essential for maintaining the genetic stability of cells, and any dysregulation may lead to cellular dysfunction or even trigger the development of cancer [16].

Recent studies have shown that ISO regulates the cell cycle through various mechanisms and significantly inhibits the proliferation of tumor cells. Zhang et al. found that ISO significantly inhibited the transformation of **bladder cancer** cells from the G0/G1 phase to the S phase and inhibited the formation of tumor spheres, which effectively inhibited cell proliferation [17]. Yang et al. further confirmed that ISO significantly inhibited the proliferation and migration of **breast cancer** cells (MCF 7/ADR) in vivo and ex vivo, and enhanced the antitumor activity of adriamycin-resistant breast cancer cells. ISO blocks cells at the G2/M phase [18]. In **oral squamous cell carcinoma** cells cell cycle arrest in the G2/M phase is caused by ISO inhibiting the expression of the cell cycle proteins cyclin B1 and CDC 2 [19]. Wang et al. also found that cell growth was arrested in the advanced **pancreatic cancer** cell line, PANC-1, after treatment with ISO. Further mechanistic studies revealed that ISO inhibits the growth of advanced pancreatic cancer cells by down-regulating cell cycle protein A and causing S-phase blockade [20]. These findings provide an important theoretical basis for ISO as a potential antitumor drug and lay the foundation for its further exploration as an antitumor therapy.

### 3.2. ISO Inhibits Cancer Cell Proliferation by Regulating the PI3K/AKT Pathway

The PI3K/AKT/mTOR signaling pathway is a key intracellular signaling pathway that plays a central role in the regulation of biological processes such as cell growth, proliferation, metabolism, and survival. When growth factors bind to their receptor tyrosine kinases (RTK), PI3K is activated and produces phosphatidylinositol-3,4,5-trisphosphate (PIP3), which is further recruited to and activated by protein kinase B, which activates the mammalian target of rapamycin, thereby affecting the activity of downstream effector molecules [21]. Dysregulation of the PI3K/AKT/mTOR pathway is associated with the progression of several diseases, including diabetes [19], autoimmune diseases [22], and tumors [23]. In tumor cells, aberrant activation of PI3K, AKT, and mTOR, as well as the deletion of phosphatase and tensin homolog (PTEN), can promote tumorigenesis. Due to the frequent activation of the PI3K/AKT/mTOR pathway in a variety of tumor types, it has become an important target for cancer therapies, and a variety of drugs targeting PI3K, AKT, or mTOR are currently on the market or under development [23].

Recent studies have shown that the inhibitory mechanisms of ISO on the proliferation of malignant tumor cells are complex and diverse, involving multiple signaling pathways and molecular mechanisms, among which the PI3K/AKT/mTOR signaling pathway plays an important role in the proliferation of many types of cancer cells. ISO significantly inhibited the proliferation and metastasis of **gallbladder cancer** cells and promoted apoptosis of gallbladder cancer cells by inhibiting the PI3K/AKT signaling pathway [24]. Interestingly, ISO not only showed significant antitumor activity in gallbladder cancer but also inhibited cell proliferation in **prostate cancer**. Cai et al. showed that ISO induced the mesenchymal–epithelial transition (MET) and inhibited matrix metalloproteinase (MMP) in a concentration-dependent manner. MET, and inhibited the overexpression of matrix metalloproteinase 2 (MMP-2) and MMP-9, thereby inhibiting cell migration and invasion. In addition, ISO significantly down-regulated the expression of phosphorylated PI3K (*p*-PI3K), phosphorylated Akt (*p*-Akt), and phosphorylated mTOR (*p*-mTOR) in two types of cancer cells, revealing a potential mechanism of action by which ISO may act as an inhibitor of the PI3K-Akt-mTOR pathway [25]. Similar results were also obtained in studies of **gastric cancer**. Li et al. found that ISO was able to block the PI3K-AKT-mTOR signaling pathway in a hypoxic environment and significantly inhibited autophagy and proliferation of gastric cancer cells in a hypoxic environment [26]. Akt not only regulates cancer cell proliferation via mTOR but also plays an important role in PPARγ signaling. It was found that ISO down-regulated the expression of PPARγ, PTEN, AKT, and CA9 proteins in the PPARγ/PTEN/AKT signaling pathway in bladder cancer cells, inhibited the activity of proliferation-related proteins, and thus, inhibited the occurrence and progression of bladder cancer [17]. ISO inhibits the activity of the PI3K/AKT/mTOR signaling pathway through multiple mechanisms, thereby inhibiting the proliferation, migration, and invasion of a wide range of cancer cells. Those findings provide an important theoretical basis for ISO as a potential antitumor drug and offer new insights for the development of novel anticancer strategies targeting the PI3K/AKT/mTOR pathway.

### 3.3. ISO Inhibits Cancer Cell Proliferation by Regulating the MAPK Pathway

The MAPK (mitogen-activated protein kinases) signaling pathway is a series of highly conserved intracellular signaling pathways that respond to a wide range of extracellular stimuli and regulate biological processes such as cell growth, differentiation, proliferation, and apoptosis. The main members of the MAPK family include the extracellular signal-regulated kinase (ERK), c-Jun amino-terminal kinase (JNK), and p38 pathways. These pathways play key roles in a variety of biological processes such as gene expression, cytoskeletal reorganization, and cell proliferation [27]. As the MAPK pathway plays an important role in tumorigenesis and progression, drug development targeting this pathway has become a key area for the treatment of related diseases.

In recent years, ISO has attracted widespread attention for its anticancer activity. It has been reported that ISO can exert anticancer effects by targeting MAPK14 and regulating the MAPK/mTOR signaling pathway or ERK/MAPK pathway [19,28]. In addition, a study by Chen Zq et al. further explored the active ingredients and targets of the Chinese medicine, Yin Chen Artemisia Decoction (Yinchenhao Decoction, YCHD), against cholangiocarcinoma. By screening the Traditional Chinese Medicine Systematic Pharmacology Database and Analysis Platform (TCMSP), quercetin, kaempferol, β-sitosterol, ISO, and stigmasterol were found to be the primary active compounds in YCHD. That study also clearly indicated that MAPK1, AKT1, IL6, TP53, and VEGFA were the main targets of those compounds, thus revealing the key mechanisms by which ISO-containing drugs exhibit anticancer effects [29].

### 3.4. ISO Inhibits Cancer Cell Proliferation by Regulating Apoptosis-Related Pathways

**Figure 2:**
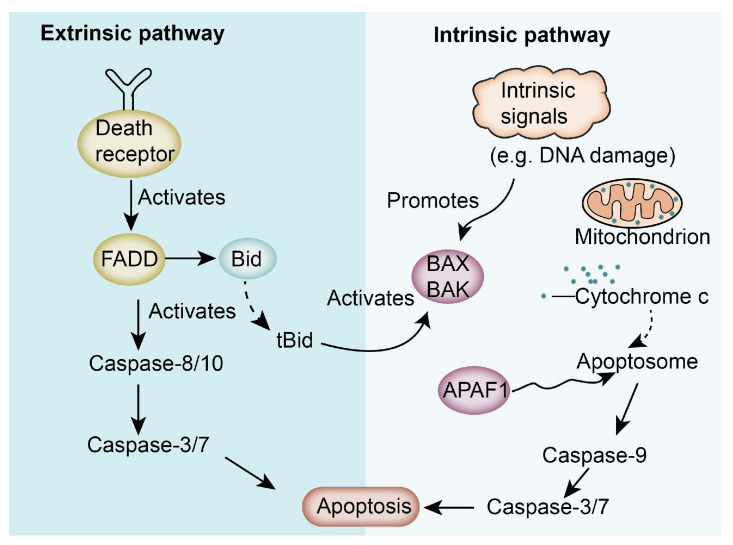
The intrinsic and extrinsic apoptotic pathways.

Abnormalities in apoptotic pathways play a crucial role in tumor development and progression. Tumor cells resist apoptosis by modulating the regulatory mechanisms of apoptosis, making tumor therapies targeting apoptosis-related mechanisms a key research topic (Figure 2).

Apoptosis, also known as programmed cell death, is an orderly, controlled death process in the cellular life cycle. Apoptosis can occur through a variety of pathways, which are mainly divided into intrinsic and extrinsic pathways. The intrinsic pathway mainly involves mitochondria and endogenous apoptotic signals. When the internal environment of the cell changes, such as due to DNA damage, oxidative stress, or the effects of drugs, the mitochondrial membrane permeability increases, resulting in the release of cytochrome C and other apoptotic factors from the mitochondria into the cytoplasm. Cytochrome C binds to apoptotic protease activator (Apaf-1) to form an apoptosome, which in turn activates caspase-9, initiating a caspase cascade that leads to apoptosis [30]. ISO can induce cell death by activating multiple apoptotic pathways. For example, M. Antunes-Ricardo et al. found that ISO reduced tumor growth, myeloperoxidase activity, and total cholesterol levels in a mouse colon cancer (HT-29 RFP) xenograft model by upregulating cleaved caspase-9, Hdac 11, and Bai 1 protein [31]. In gastric cancer studies, ISO induced apoptosis of cancer cells by inducing the release of cytochrome C from mitochondria into the cytoplasm via the mitochondrial apoptotic pathway [32].

Apoptosis can also occur via an extrinsic pathway that involves death receptors, such as members of the tumor necrosis factor receptor (TNF-R) family. When bound to their corresponding ligands (e.g., Fas ligand, TNF-α), those receptors trigger the aggregation of death domains on the cell surface. This aggregation activates caspase-8 or caspase-10, which in turn activates downstream effector caspases (e.g., caspase-3, caspase-6, and caspase-7), leading to apoptosis [30]. It has been reported that ISO can further promote apoptosis by promoting the accumulation of intracellular ROS. That finding is consistent with the study of Li et al. who found that ISO treatment of gastric cancer cells (AGS-1 and HGC-27) initiated the activation of the caspase-3 cascade and increased the expression of Bax/Bcl-2 and cytochrome C, and activated caspase-3 cleavage and poly (ADP-ribose) polymerase (PARP)), leading to a reduction in mitochondrial membrane potential and the accumulation of ROS. In addition, ISO not only has a pro-apoptotic effect on human gastric cancer cells, but also shows a good pro-apoptotic effect in human melanoma cells and human bladder cancer cells, which increases the expression of Bax and caspase 3 proteins, and reduces the expression of Bcl-2 protein [33].

Boshra’s study intriguingly investigated how ISO interacts with caspase 2. The researchers identified ISO and its glycoside derivatives, such as isorhamnetin-O-hexoside and isorhamnetin-O-rutinoside, as the primary active components in the methanolic extract of Saussurea costus roots, using UPLC/T-T-TOF-MS/MS for analysis. In a model of acetaminophen-induced liver damage, there was a notable increase in plasma caspase 2 activity, indicating an abnormal activation of the apoptotic pathway. However, pretreatment with MESC significantly lowered caspase 2 levels, suggesting that it offers protection to the liver by modulating apoptotic signaling. As a key flavonoid in MESC, ISO may influence caspase 2 activity through two main mechanisms: first, by directly inhibiting the enzymatic activity of caspase 2 and blocking its downstream apoptotic cascade; and second, by indirectly decreasing caspase 2 activation through the upregulation of hepatocyte nuclear factor HNF-1α and Sirtuin-1, which enhances the cell’s antioxidant and anti-inflammatory capabilities. Furthermore, MESC’s negative regulation of miRNA-34a and miRNA-223 may also contribute to the inhibition of the caspase 2-related apoptosis signaling pathway. Thus, ISO serves as a multi-target regulator, playing a significant role in the anti-apoptotic effects of MESC, with its mechanisms likely linked to the antioxidant, anti-inflammatory, and epigenetic regulatory properties of flavonoids [34].

The endoplasmic reticulum is the primary site of protein synthesis and folding. When protein folding is faulty or the endoplasmic reticulum is stressed, the unfolded protein response (UPR) is activated. If the stress persists, the UPR is converted into a pro-apoptotic signal. Endoplasmic reticulum stress can activate JNK and C/EBP homologous protein (CHOP), which promote apoptosis [30]. Ye et al. showed that in endometrial cancer treatment, ISO promotes endoplasmic reticulum stress-related pathways through the activation of endogenous mitochondrial apoptosis pathways and exogenous death receptor pathways. Those actions then activate corresponding UPR response markers and induce apoptosis in endometrial cancer cells. ISO also affects the expression of MMP-2 and MMP-9 related proteins in vitro and in vivo, and ultimately inhibits tumor metastasis. Therefore, ISO could be a promising drug for the treatment of endometrial cancer [35].

Canine cancers share similarities with human cancers in terms of disease characteristics, subtype distribution, and treatment strategies. Similar to human breast cancer, different subtypes, such as estrogen receptor (ER)/progesterone receptor (PR)-positive, human epidermal growth factor receptor 2 (HER2)-positive, and triple-negative breast cancer (TNBC), have been observed in canine breast cancers. Both canine mammary tumor (CMT) and human breast cancer (HBC) occur in complex microenvironments that influence the growth and metastasis of cancer cells. CMT and HBC show a high degree of similarity in immune infiltration and immune escape mechanisms. In addition, CMT exhibits abnormalities in many genes associated with HBC, such as the activation of the PI3K/AKT/mTOR signaling pathway [36]. A previous study by Mei et al. found that ISO could promote apoptosis in canine breast cancer by down-regulating the epidermal growth factor receptor (EGFR)/signal transducer and activator of transcription 3 (STAT3)/programmed death ligand 1 (PD-L1) signaling pathway to promote apoptosis [37]. In addition, human and canine triple-negative breast cancer can activate the same signaling pathway [38].

### 3.5. ISO Inhibits Cancer Cell Proliferation by Regulating Autophagy-Related Pathways

The role of autophagy in cancer cells is extremely complex and it exhibits a remarkable duality in different cancer stages and microenvironments. Autophagy can either play an inhibitory role or may promote the growth and survival of cancer cells. This duality makes autophagy a hot area in cancer research. In the early growth stage of tumors, autophagy suppresses tumorigenesis by removing damaged organelles and misfolded proteins and maintaining genomic stability. However, during tumor progression, the role of autophagy changes, potentially shifting to a promotional role for cancer cells by degrading intracellular structures and releasing energy or molecules for their use [39].

Hypoxia is a common feature of the tumor microenvironment, and several studies have shown that the hypoxic microenvironment promotes tumor cell survival, motility, and metastasis through the activation of adaptive transcriptional programs, thereby driving cancer progression. Hypoxia can lead to organelle damage, which in turn activates cellular autophagy. A hypoxic environment induces adaptive autophagy in tumor cells, and blocking autophagy inhibits the growth of gastric cancer cells. When autophagy is inhibited, the accumulation of damaged organelles accelerates cell death [39]. Li et al. found that ISO could effectively inhibit autophagy in gastric cancer cells grown in a hypoxic environment, thereby suppressing tumor development and progression. Specifically, the expression levels of autophagy marker proteins LC3B and Beclin 1 were significantly reduced, while the expression of equestosome 1 (P62/SQSTM1) was increased in gastric cancer cells (MKN-45) treated with ISO (P62 is a stress-induced intracellular protein, and, as a multifunctional protein for selective autophagy, the expression level of P62 is negatively correlated with autophagic activity) [26].

The inhibitory effect of ISO on the growth of human lung cancer cells (A549) was further investigated in a study by Ruan et al. The results showed that autophagy inhibition enhanced ISO-induced mitochondria-dependent apoptosis in non-small cell lung cancer cells. In that study, the proliferation and colony formation abilities of lung cancer cells (A549) treated with ISO were significantly inhibited. In addition, ISO treatment induced apoptotic cell death in A549 cells, and the apoptotic process exhibited significant time and dose dependence. In-depth studies revealed that apoptosis of A549 cells was mediated by a mitochondria-dependent pathway, which was caused by alterations in mitochondrial membrane potential, the release of cytochrome C, and the activation of caspases. Notably, ISO treatment also induced the formation of autophagosomes as well as light chain 3-II protein in A549 cells [40].

A study by Kirilowii et al. also found that ISO inhibited cell proliferation in a variety of human cancer cell lines (MDA-MB-231, MCF-7, A549, SMMC-7721, Eca109, HEB, and MCF-10A cells). ISO caused human breast cancer cell cycle arrest in the G2/M phase and induced apoptosis and autophagy. Molecular docking results showed that ISO docked in the ATP-binding pocket of PI3Kγ. Importantly, ISO treatment significantly reduced the levels of PI3Kγ-p110, *p*-PI3K, *p*-AKT, *p*-mTOR, *p*-p70S6K, and *p*-ULK [41].

### 3.6. ISO Inhibits Cell Proliferation by Modulating the Tumor Microenvironment and Non-Coding RNA-Related Pathway

In the field of tumor immunotherapy, the application of ISO and its potential mechanisms of action have received substantial attention. A study by Liu et al. developed a dual-functional mesoporous silica nanoparticle (HMSN-ISO@ProA-PD-L1 Ab), which effectively modulated the tumor immune micro-environment by combining ISO and human PD-L1 antibodies. Experimental results showed that the nanoparticle significantly reduced the levels of myeloid-derived suppressor cells (MDSCs) in the tumor microenvironment and promoted the infiltration of T cells within the tumor, thus exerting a synergistic antitumor effect [42].

Chen et al. conducted a systematic study of the active ingredients in the traditional Chinese medicine Yin Chen Artemisia Decoction (Yinchenhao Decoction) using a network pharmacological approach to investigate its inhibitory effect on cholangiocarcinoma. ISO was an important active compound in the formulation. Molecular docking experiments further revealed that ISO has high affinity for several key targets, including AKT-1, IL-6, MAPK-1, TP-53, and VEGFA. Such targets affect KKU-M213 cell growth and survival by regulating microRNAs in the MAPK signaling pathway and the PI3K-Akt signaling pathway, which in turn affects cancer cell growth and survival [29].

### 3.7. ISO Inhibits Cell Proliferation by Modulating the Pyroptosis Pathway

Pyroptosis is a form of programmed cell death whose core mechanism involves the activation of multiple signaling pathways. In the classical pathway, the inflammasome activates caspase-1, which triggers cellular pyroptosis. In addition, lipopolysaccharide activates caspase-4/5/11, which cleave gasdermin D (GSDMD), forming pores in the cell membrane. Meanwhile, granzyme A (GZMA) and granzyme B (GZMB) enter the target cell via perforin and cleave gasdermin family members, GSDMB and GSDME, respectively, forming cell membrane pores. The formation of pores leads to the release of cellular contents and triggers an inflammatory response.

**Figure 3:**
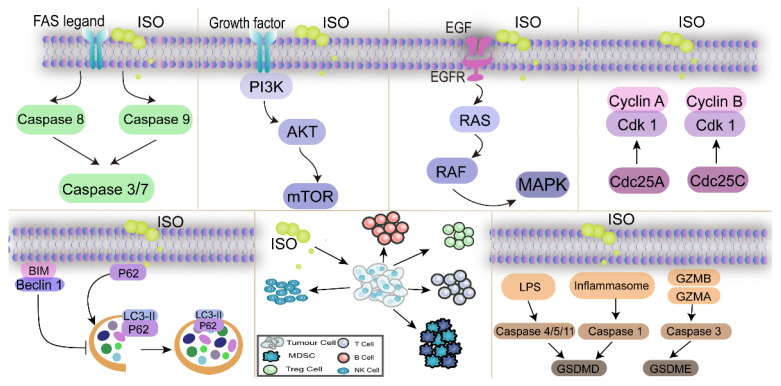
Mechanism of action of ISO against cancers. The arrows represent the role of promotion in the diagram.

Pyroptosis plays an important role in defending against multiple diseases such as cancer. With increasing research on the pyroptosis pathway in recent years, its potential application in disease therapy has gradually emerged, providing new avenues for the treatment of related diseases [43]. Wendlocha et al. systematically investigated the inhibitory effects of several flavonols, including ISO, on cancer. That study evaluated the potential of such compounds in targeting different types of cancer cell death pathways and found that flavanols could induce cell death via multiple mechanisms, including pyroptosis [44]. These findings not only help to clarify the anticancer mechanisms of flavonols such as ISO but also provide an important theoretical basis for the future development of drug regimens based on the pyroptosis pathway [44]. The mechanisms leading to the anticancer effects of ISO are summarized in Figure 3.

## 4. The Role of ISO in Targeted Therapies and Its Molecular Mechanism of Action Based on Molecular Docking

A study by Li et al. significantly inhibited autophagy and the proliferation of gastric cancer cells in a hypoxic environment by targeting PI3K and blocking PI3K-AKT-mTOR signaling. The study also observed a decrease in mitochondrial membrane potential and the promotion of apoptosis [45]. In another study, Chen et al. predicted the potential targets of ISO in cholangiocarcinoma cells using a network pharmacological approach. Further molecular docking experiments showed that ISO has good binding affinity to AKT1, IL-6, MAPK1, TP53, VEGFA, and other proteins, all with binding affinities less than −5 kcal/mol, and thus suggest that those proteins may be the direct targets of ISO [29]. However, those predictions were only made using software simulations, and experimental methods such as plasma resonance are needed to further verify whether the molecular forces between ISO and target proteins are enhanced in a concentration-dependent manner.

In a screen of active ingredients in traditional Chinese medicine formulas, researchers predicted the targets of the antigastric cancer agent, Guiqi Baijia, and analyzed the potential key compounds, key targets, and key signaling pathways. The results showed that the active ingredients of Guiqi Baijia could target 33 proteins in gastric cancer cells. Signaling pathway analysis indicated that the PI3K-AKT signaling pathway may play a key role in gastric cancer [46]. Based on those findings, Li et al. further selected HER-2 and PD-L1 for molecular docking simulations. The results showed that 385 and 189 compounds had high docking scores with HER-2 and PD-L1 proteins, respectively. Subsequently, the authors selected six pairs of compounds to perform microcalorimetric thermophoretic movement experiments with HER2 and PD-L1 to characterize their binding abilities. The experiments showed that ISO had the highest binding affinity to PD-L1, with a KD value of 667 nmol/L [45] and demonstrated that ISO can exert antitumor effects by targeting key proteins in cancer cells. Those findings also not only provide a theoretical basis for the application of ISO as a potential anticancer drug, but also provide a new research direction for antitumor therapeutic strategies based on herbal formulas.

## 5. Advantages of ISO in Clinical Use

### 5.1. Limitations of Chemotherapy and the Potential of ISO Combination Therapy

Chemotherapy is an important cancer treatment, but its clinical application has many limitations. While killing cancer cells, chemotherapeutic drugs also cause damage to normal cells, triggering systemic toxic side effects such as bone marrow suppression, nausea, vomiting, and hair loss. In addition, some cancers are not sensitive to chemotherapeutic drugs or are prone to drug resistance. The chemotherapeutic process is usually lengthy, and patients are hospitalized repeatedly for treatment, which not only imparts physical pain to patients but also causes an economic burden and psychological pressure. In contrast, the combination of plant-derived small molecules has unique advantages. These compounds have multiple sites of action, and can not only be used as adjuvants to chemotherapy but may also reduce the resistance of chemotherapeutic drugs, thus providing more effective treatment options for cancer patients. The combined application of ISO with cisplatin shows good prospects in antitumor therapy [47].

### 5.2. The Use of ISO in Mitigating the Toxic Effects of Chemotherapy

ISO reduces chemotherapy toxicity. A study by Wang et al. investigated the potential of ISO to attenuate cisplatin-induced acute kidney injury. Cisplatin is a widely used chemotherapeutic agent for the treatment of many solid tumors, but its nephrotoxicity limits its clinical application. ISO is a natural flavonoid with pharmacological effects such as anti-inflammatory and antioxidant activities. In vitro experiments showed that ISO significantly inhibited the toxic effects of cisplatin on human renal tubular epithelial cells, and had a significant inhibitory effect on cisplatin-induced apoptosis and inflammatory responses. In a mouse model of acute kidney injury induced by a single intraperitoneal injection of 20 mg/kg cisplatin, oral administration of ISO 2 days before or 2 h after cisplatin injection significantly improved renal function and tubular injury. Transcriptomic RNA-seq analysis of mouse kidney tissues showed that ISO treatment protected against cisplatin-induced nephrotoxicity via PGC-1α-mediated fatty acid oxidation. ISO significantly enhanced lipid clearance, ATP levels, and the expression of PGC-1α and its downstream target genes, PPARα and CPT1A, which were reduced by cisplatin. Those findings suggest that ISO has potential clinical application for the management of cisplatin-induced nephrotoxicity [46].

### 5.3. Enhancement of Chemotherapeutic Drug Sensitivity by ISO

In a study by Li et al., ISO was found to enhance the effects of chemotherapy in TNBC. As a natural compound, ISO has received much attention for its potential to enhance the sensitivity of chemotherapeutic drugs. Studies have shown that ISO enhances the sensitivity of cancer cells to chemotherapeutic drugs via multiple mechanisms. In a mouse model, chloroquine (CQ) or ISO alone resulted in only a mild increase in survival time (36 or 39 days, *n* = 10). However, the combination of CQ and ISO significantly prolonged the median survival time of mice to 62 days. Hu et al. further determined the effect of the CQ/ISO combination on tumor growth in TNBC xenografts. Those authors showed that CQ alone had no significant effect on tumor growth, while ISO treatment alone moderately inhibited tumor growth. However, the combination therapy significantly inhibited the growth of human triple-negative breast cancer. Mechanistic studies revealed that CQ inhibited autophagy/mitochondrial autophagy and selectively enhanced ISO-induced mitochondrial division and apoptosis in TNBC cells. However, that effect was not observed in estrogen-dependent breast cancer cells. The combination of CQ/ISO-induced apoptosis is associated with the activation of CaMKII and Drp1 (S616) in a TNBC xenograft mouse model. It was also found that the ROS-mediated CaMKII/Drp1 signaling pathway plays an important role in the regulation of mitochondrial division and apoptosis induced by the combination of CQ/ISO [47]. These findings suggest that ISO is promising for further development as a new chemotherapeutic agent. In addition, the combination of ISO with classical autophagy/mitochondrial autophagy inhibitors may become a new strategy for the treatment of TNBC. As a multi-targeted natural compound, ISO shows promising applications in alleviating the toxic side effects of chemotherapy and enhancing the sensitivity of chemotherapeutic drugs. This multi-target combination therapy strategy also provides a new avenue for the individualized treatment of tumors. Future clinical studies will further validate the safety and efficacy of ISO in combination with other antitumor drugs, thus providing additional therapeutic options for tumor patients.

### 5.4. ISO Has a Protective Effect on Normal Cell

During chemotherapy and radiotherapy, the protection of normal cells from damage is one of the key factors that improves the therapeutic effect. ISO exhibits good protection of normal cells and can effectively reduce the toxicity of chemotherapeutic drugs on normal cells.

Recent studies investigated the cytotoxic effects of ISO and its derivative ISO-3-glucuronide (I3G) in human breast cancer cells (MCF-7) to reveal their tumor-suppressor mechanisms and structure–function relationships. The results showed that quercetin (Que), ISO, and I3G all inhibited the growth of MCF-7 cells in a dose-dependent manner. The order of the potency of their cytotoxic effects was Que > ISO > I3G. Those compounds also mediated cell cycle arrest, mainly in the S-phase, and the number of cells in subsequent G0/G1 and G2/M phases was reduced. When treated at a concentration of 100 μM for 48 h, 70.8%, 68.9%, and 49.8% of MCF-7 tumor cells entered early apoptosis. Moreover, apoptosis induced by quercetin, ISO, and ISO-3-glucuronide was accompanied by a significant increase in intracellular ROS. Those results suggest that quercetin, ISO, and ISO-3-glucuronide exert cytotoxic effects in MCF-7 cells through the ROS-dependent apoptotic pathway.

To further evaluate the effects of ISO and its derivatives on normal cells, Wu et al. used normal breast cells (H184B5F5/M10) as the test group and examined the effects of the same concentrations of ISO and ISO-3-glucuronide on cell proliferation. The results revealed that both ISO and ISO-3-glucuronide failed to inhibit growth or exhibit cytotoxicity in normal breast cells [48]. That finding suggests that ISO and its derivatives inhibit the growth of tumor cells while exhibiting good protective effects in normal cells. The findings provide new perspectives on the application of ISO in cancer therapy, highlighting its potential as an adjuvant to chemotherapy and radiotherapy. ISO not only enhances the sensitivity of tumor cells to chemotherapeutic drugs but also effectively protects normal cells and reduces the side effects of treatment. Those properties highlight ISO as a novel therapeutic adjuvant with clinical applications. Future studies will further explore its specific mechanisms of action and clinical applications in cancer therapy.

## 6. Pharmacokinetics and Safety Assessment of ISO

The properties of a drug directly affect its efficacy and safety. Pharmacokinetics (PK) is the study of drug absorption, distribution, metabolism, and excretion in the body. Therefore, pharmacokinetic and safety assessments are essential to the drug development process. In recent years, with the continuous advancement of drug development technologies, pharmacokinetic research methods have been developed to better understand the behavior of drugs under different physiological conditions.

ISO has extremely low water solubility (<3.5 μg/mL) which limits its potential for clinical applications. To improve the aqueous solubility of ISO for intravenous administration, Giovanna Rassu et al. used polyvinylpyrrolidone (PVP 10) and benzalkonium chloride as solubilizing agents to improve the solubility of ISO by ~600-fold to reach 2.1 mg/mL. Those researchers administered the solution intravenously to rats at a dose of 0.5 mg/kg and its pharmacokinetic properties were examined in detail. The results showed that the pharmacokinetic behavior of ISO was consistent with a two-compartment model, exhibiting a rapid initial distribution phase (t1/2α: 5.7 ± 4.3 min) and a slow elimination phase (t1/2β: 61 ± 47.5 min) [49]. That study successfully improved the bioavailability of ISO and laid the foundation for its further clinical application.

## 7. ISO Can Be Prepared in Different Forms

ISO can be prepared in many different forms to enhance its potential application in disease treatment. In recent years, researchers explored the mechanism of action and clinical prospects of ISO for the treatment of various diseases through different formulation strategies and pharmacological approaches.

Liu et al. found that ISO can inhibit the growth of hepatocellular carcinoma cells by targeting the deubiquitinating enzyme USP7 and promoting the ubiquitin-dependent degradation of the transcription factor, YY1. Based on those findings, Liu et al. combined ISO with PD-L1 antibodies to prepare the nanoparticles, HMSN-ISO@ProA-PD-L1 Ab. Those nanoparticles can specifically target hepatocellular carcinoma cells and play an important role in the controlled release of ISO. Experimental results showed that HMSN-ISO@ProA-PD-L1 Ab nanoparticles inhibited the growth of hepatocellular carcinoma cells (Hepa 1–6 graft tumors) better than that of the groups treated with PD-L1 antibody or ISO alone. In addition, the nanoparticles significantly reduced the levels of MDSCs in the tumor microenvironment and promoted the infiltration of T cells within the tumor, thereby improving the tumor immune microenvironment [42].

Zhang et al. prepared Danlu tablets, a traditional Chinese medicine containing ISO and systematically analyzed its main mechanism of action during the treatment of non-small cell lung cancer using network pharmacology, molecular docking, and molecular dynamics. The results showed that Danlu tablets exerted antitumor effects through multiple pathways, including the inhibition of tumor cell proliferation, induction of apoptosis, and modulation of immune responses. Validated using in vitro experiments, this study provided useful predictive information for clinical treatment and had a positive impact on the development of new drugs and the modernization of traditional Chinese medicines via drug repositioning [50].

Wang et al. used Chaiqin Qingning Capsule, a traditional Chinese medicine containing ISO, in pharyngitis. In a rat pharyngitis model, the researchers investigated the antipharyngitis active ingredients and mechanism of action of Chaiqin Qingning Capsule. Chai Baicalin Qingning capsule could significantly reduce pharyngitis symptoms via multiple mechanisms, including anti-inflammatory, antioxidant, and immunomodulation activities. That study provided a new concept to explore the interventional effects of the Chai Baicalin Qingning capsule on pharyngitis and other upper respiratory tract diseases [51].

**Figure 4:**
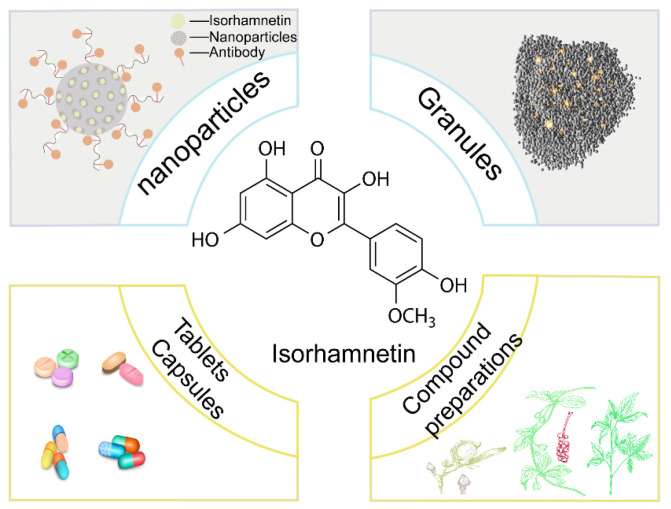
ISO in different forms.

Ju et al. prepared Chanqin granules from multiple traditional Chinese medicines (TCM) that contain ISO as the main active ingredient and explored its application for the treatment of multiple diseases, such as tumors, digestive diseases, cardiovascular diseases, respiratory diseases, and neurological diseases. Through multi-component, multi-target, and multi-pathway mechanisms of action, toad Scutellaria granules demonstrated good therapeutic efficacy and safety [52]. In addition, Chen et al. prepared Yinchenhao Decoction, a TCM containing ISO, for the treatment of cholangiocarcinoma. The results of that study showed that Yinchenhao Decoction exerts antitumor effects through multi-component, multi-target, and multi-pathway mechanisms, thus providing new options for the treatment of cholangiocarcinoma [29]. ISO can treat multiple diseases using different formulations and pharmacological approaches (Figure 4). Studies not only revealed the potential therapeutic mechanisms of ISO in different diseases but also provided an important theoretical basis and experimental support for the modernization of TCM and the development of new drugs. Future studies will further explore the safety and efficacy of ISO in clinical applications and provide additional possibilities for the development of novel therapeutic drugs.

## 8. Summary

**Figure 5:**
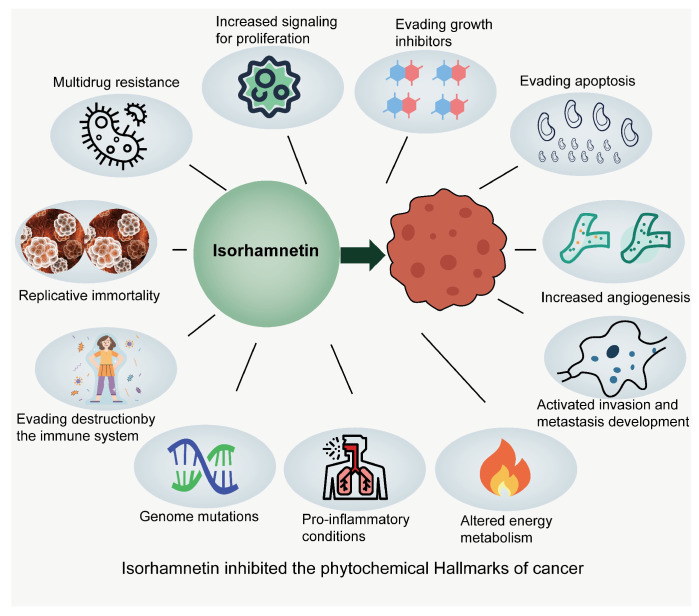
ISO inhibits the hallmarks of cancer.

In this review, we provided insight into the key findings of ISO use in malignancy research and analyzed its effects in combination with other antitumor agents. As a natural compound with multiple modes of action (Figure 5), ISO shows promising applications in antitumor research. By analyzing its role in combination chemotherapy and targeted therapy, we found that ISO not only enhances the antitumor effect of other drugs but also reduces their side effects, thus improving patient treatment compliance and quality of life.

However, despite the gradual recognition of the antitumor potential of ISO, some discrepancies exist in the results of different studies regarding its mechanism of action. Such discrepancies suggest that the mechanism of action of ISO in different tumor types and patient groups should be explored more deeply to better understand their biological bases. It is also necessary to consider the effect of the combination of ISO and other antitumor drugs on different cancers, and to verify its safety and efficacy in large clinical trials to provide a scientific basis for its clinical application.

Future research should include the following: determining the optimal administration regimen and dosage of ISO is a critical step toward its clinical application. Studies should focus on optimizing the route of administration, frequency, and dosage to ensure the effective distribution and metabolism of the drug in the body while minimizing toxic side effects. Strategies should be explored for the combination of ISO with other antitumor drugs to achieve synergistic antitumor effects. Through mechanistic studies and clinical trials, the optimal drug combinations and combined treatment protocols should be determined, which is expected to further improve the therapeutic effects.

A sound clinical translation mechanism should be established to promote the translation of basic research for clinical applications. Such activities require interdisciplinary cooperation and the integration of resources from biomedicine, pharmacology, clinical medicine, and other fields to accelerate the translation of ISO from the laboratory to the clinic. Interdisciplinary cooperation and the integration of resources and expertise from multiple fields is expected to support the clinical application of ISO. Such collaboration will not only contribute to the in-depth understanding of the mechanisms of action of ISO but will also provide additional strategies for its application in the treatment of malignant tumors.

As a natural compound with multiple modes of action, ISO is promising for the treatment of malignant tumors. Although its antitumor potential has been recognized, future research is needed to explore its mechanism of action in different tumor types and patient groups, and to verify its safety and efficacy in large-scale clinical trials. By optimizing the dosing regimen, exploring the combination application strategy, and establishing a clinical translation mechanism, we expect ISO to play a greater role in the treatment of malignant tumors, and ultimately improve the prognosis and quality of life of patients.

## Abbreviations

The following abbreviations are used in this manuscript:

ACE2	angiotensin-converting enzyme 2
AKT	protein kinase B
Apaf-1	apoptotic protease activator
Cdc25A	cell division cycle 25A
Cdc25C	cell division cycle 25C
Cdk1	cyclin dependent kinase 1
CMT	canine mammary tumor
EGF	epidermal growth factor
EGFR	epidermal growth factor receptor
ER	estrogen receptor
ERK	extracellular signal-regulated kinase
FAS	fatty acid synthase
GSDMD	gasdermin D
GSDME	gasdermin E
GZMA	granzyme A
GZMB	granzyme B
HBC	human breast cancer
HER2	human epidermal growth factor receptor 2
ISO	isorhamnetin
JNK	c-Jun amino-terminal kinase
LC3-II	microtubule-associated-proteinlight-chain-3
LPS	lipopolysaccharide
MAPK	mitogen-activated protein kinases
MDSCs	myeloid-derived suppressor cells
MESC	saussurea costus
MET	mesenchymal-epithelial transition
MMP	matrix metalloproteinase
mTOR	mammalian target of rapamycin
PD-L1	programmed death ligand 1
PI3K	phosphoinositide-3 kinase
PR	progesterone receptor
PTEN	phosphatase and tensin homolog
ROS	reactive oxygen species
STAT3	signal transducer and activator of transcription 3
TNBC	triple-negative breast cancer
TNF-R	tumor necrosis factor receptor
